# Strong Manual Acupuncture Stimulation of “Huantiao” (GB 30) Reduces Pain-Induced Anxiety and p-ERK in the Anterior Cingulate Cortex in a Rat Model of Neuropathic Pain

**DOI:** 10.1155/2015/235491

**Published:** 2015-12-03

**Authors:** Xiao-mei Shao, Zui Shen, Jing Sun, Fang Fang, Jun-fan Fang, Yuan-yuan Wu, Jian-qiao Fang

**Affiliations:** Department of Neurobiology & Acupuncture Research, The Third Clinical College, Zhejiang Chinese Medical University, Hangzhou 310053, China

## Abstract

Persistent neuropathic pain is associated with anxiety. The phosphorylation of extracellular signal-regulated kinase (p-ERK) in the anterior cingulate cortex (ACC) plays an important role in pain-induced anxiety. Acupuncture is widely used for pain and anxiety. However, little is known about which acupuncture technique is optimal on pain-induced anxiety and the relationship between acupuncture effect and p-ERK. The rat model was induced by L5 spinal nerve ligation (SNL). Male adult SD rats were randomly divided into control, SNL, strong manual acupuncture (sMA), mild manual acupuncture (mMA), and electroacupuncture (EA) group. Bilateral “Huantiao” (GB 30) were stimulated by sMA, mMA, and EA, respectively. The pain withdrawal thresholds (PWTs) and anxiety behavior were measured, and p-ERK protein expression and immunoreactivity cells in ACC were detected. PWTs increased significantly in both sMA and EA groups. Meanwhile, anxiety-like behavior was improved significantly in the sMA and mMA groups. Furthermore, the overexpression of p-ERK induced by SNL was downregulated by strong and mild manual acupuncture. Therefore, strong manual acupuncture on bilateral “Huantiao” (GB 30) could be a proper therapy relieving both pain and pain-induced anxiety. The effect of different acupuncture techniques on pain-induced anxiety may arise from the regulation of p-ERK in ACC.

## 1. Introduction

Anxiety and depression often coexist with persistent pain [1–4]. In humans, patients with persistent pain frequently suffer from a series of aversive emotions including anxiety, fear, depression, loneliness, and misanthropy, which can be more distressing than the pain itself [5].

In clinical studies, Chinese acupuncture has been practiced in many cultures and is nowadays widely used to relieve pain all over the world [6–9]. In 1996, the World Health Organization conference in Milan suggested 64 indications for acupuncture, including many psychiatric disorders such as cardiac neurosis, depression, and schizophrenia. Manual acupuncture (needling using manual stimulation) and electroacupuncture (EA, needling with electrical stimulation) are two common methods of acupoint stimulation. Both are applied clinically for the treatment of chronic pain and various mental disorders [10, 11]. Furthermore, manual acupunctures were divided into mild manual acupuncture (mMA) and strong manual acupuncture (sMA) on the basis of strength for needling manipulation, defined as reinforcing and reducing methods, respectively, in traditional Chinese medicine. But studies on the distinct effects of the three methods (EA, mMA, and sMA) are lacking. In last decades, preclinical and clinical researches have demonstrated that MA and EA are, respectively, effective for neuropathic pain [12–15] and anxiety [16–18]; however, it is unknown whether they have an effective role for neuropathic pain-induced negative mood.

Several studies have reported that persistent pain in humans is associated with changes in brain anatomy [19] and suggest that activation of the ACC has been found to be associated with the affective dimension of pain [20–23]. No part of the cingulate cortex is activated only by noxious stimulation, although there may be small aggregates of purely nociceptive neurons [24]. Therefore, it is important to determine the functional significance of changes in the ACC when studying persistent pain-induced anxiety and other mood disorders.

Accumulating evidence has shown that extracellular signal-regulated kinase (ERK), a family member of mitogen-activated protein kinases (MAPKs), in the ACC is activated in the chemical inflammatory pain or neuropathic pain model [25, 26] and suggested that pain-induced anxiety is regulated by the ERK activation in the ACC after incision [27]. Moreover, inhibition of ERK1/2 activation in ACC after acetic acid injection by subcutaneous injection of the mitogen-activating extracellular kinase (MEK) inhibitor, SL327, attenuates visceral pain-induced anxiety-like behavior [28]. All of these data demonstrate that ERK activity in the ACC may be an important hub for various types of pain-induced anxiety and thus constitutes a critical target for revealing the underlying mechanism.

In the present work, we hypothesized that mMA, sMA, and EA have differential effects on pain-induced anxiety and that the cellular mechanism underlying such anxiety involves ERK phosphorylation in the ACC. To test these hypotheses, we used the L5 spinal nerve ligation (SNL) rat model of persistent pain to assess the changes in pain-induced anxiety and phosphorylated- (p-) ERK levels in the ACC and to investigate the effect of mMA, sMA, and EA on these measures (Table 1).

## 2. Methods

### 2.1. Subject

Male adult Sprague-Dawley rats, about 70 days old (220–250 g), were obtained from the Experimental Animal Center of Zhejiang Chinese Medical University. The animals were housed in groups of five in plastic cages with soft bedding at the University Animal Care facility, with an artificial 12/12 h light-dark cycle (lights on at 8 a.m.). Animals received food and water ad libitum with a constant room temperature of 23–25°C and a relative humidity of 40–70%. Before experimental manipulations, the rats were given 1 week to adjust to their new surroundings. All animal procedures performed in this work followed guidelines in accordance with the Regulations for the Administration of Affairs Concerning Experimental Animals and were approved by the Animal Care and Welfare Committee of Zhejiang Chinese Medical University, Zhejiang, China.

### 2.2. Surgery for Neuropathic Pain Model

The rats were anesthetized with 7% (w/v) choral hydrate (5 mL/kg, intraperitoneally). Each rat was placed on a heated surgical platform at a constant temperature at 37°C in the prone position, and the right paraspinal muscles were separated from the spinous processes at the L4-S2 levels. The L6 transverse process was carefully removed with a small rongeur to identify the L4-L5 spinal nerves visually. Once enough length of the L5 spinal nerve was freed from the adjacent structure, a piece of 6/0 silk thread was placed around the L5 spinal nerve and pulled tightly to interrupt all axons in the nerve. On completion of the operation, hemostasis was confirmed and the muscles were sutured in layers using silk thread. Finally, animals were placed in a new cage with warm bedding until complete recovery from anesthesia. After the operation, the rats showed no motor deficits except a mild inversion of the ipsilateral hind paw with slightly ventroflexed toes.

### 2.3. EA, sMA, and mMA Procedures

EA, sMA, and mMA treatments were applied, respectively, to bilateral acupoints “Huantiao” (GB 30, located at the junction of the lateral 1/3 and medial 2/3 of the distance between the great trochanter of the femur and the last sacral vertebrae) and “Yanglingquan” (GB 34, located in the depression anterior and inferior to the small head of the fibula) (Figure 5) every other day from 3 to 11 days after SNL. In the EA stimulation group, inserted needles (0.3 mm in diameter and 10 mm in depth) on bilateral “Huantiao” and “Yanglingquan” were attached to the output terminals of the Hans Acupoint Nerve Stimulator (HANS 200E, Huawei Co., Ltd., Beijing, China). The EA parameters were set as follows: constant square wave current output (pulse width: 0.6 ms at 2 Hz, 0.2 ms at 100 Hz); remaining intensities at 1 ± 0.5 mA (causing the slight vibration of muscles around acupoints); alternating frequencies of 2 Hz and 100 Hz (automatically shifting between 2 Hz and 100 Hz stimulation for three seconds each). In the sMA stimulation group, sterilized disposable stainless steel needles (0.3 mm in diameter) on bilateral “Huantiao” were inserted to a depth of 10 mm and then twisted manually clockwise and counterclockwise (360°) for 2 min at a rate of 180 times per min, followed by an interval of 13 min with needles retained and then another 2 min twisting stimulation. Finally the needles were retained in place for 13 min before removal. In the MA stimulation group, sterilized disposable stainless steel needles (0.22 mm in diameter) on bilateral “Huantiao” were inserted to a depth of 10 mm and then twisted manually clockwise and counterclockwise (180°) for 2 min at a rate of 60 times per min, followed by an interval of 13 min with needles retained and then another 2 min twisting stimulation. Finally the needles were retained in place for 13 min before removal. The total time including twisting and retaining the needles is 30 min. In two MA groups, bilateral “Yanglingquan” simply retained the needles for 30 min without any twirling and rotating manipulation. In the whole procedure, all rats maintained relatively comfortable states without any struggling and screaming.

### 2.4. Nociceptive Behavioral Testing

Mechanical hyperalgesia confirmed the success of the SNL. The PWT was automatically measured with a dynamic plantar aesthesiometer (model 37450; Ugo Basile, Comerio, Italy). Animals were habituated to the testing surroundings daily for two consecutive days (between 9 a.m. and 12 p.m.) before baseline testing. The room temperature and humidity remained stable with a low noise level (<40 dB) during testing. Bilateral PWTs were measured before SNL and 3, 7, and 12 days after SNL. Each rat was allowed to move freely in a transparent plastic compartment of a six-compartment box with a wire mesh floor and acclimatize for 20 min before the test session. A paw-flick response was elicited by applying an increasing vertical force (increase steadily from 0 to 50 grams in 20 sec) produced by a stainless steel probe (a straight 0.5 mm diameter) which was placed underneath the mesh floor and focused on the middle of the plantar surface of the bilateral hind paws. The hind paws of each rat were measured five times with at least 1 min intervals and then averaged. All manipulations were taken by the same operator. The whole test was performed by an investigator blind to the experimental groups.

### 2.5. Anxiety-Like Behavioral Testing

Repeated exposure to test conditions may significantly decrease anxiety-like behaviors. Therefore, in the present study, anxiety-like behavior was tested only once, 12 days after SNL. Spontaneous exploratory activity was monitored using an automatic video tracking system (SMART, Panlab, Spain) and all parameters were analyzed by SMART software (version 3.0, Panlab). Test apparatus were cleaned with 70% ethanol and dried after each testing session.

The elevated zero maze consisted of two open (stressful) and two enclosed (protecting) sections opposite to each other, forming a black plastic annular platform (100 cm diameter, 25 cm width, and 50 cm above the ground). The enclosed sections had walls (30 cm high) on the inner and outer edges. At the beginning of the 5 min testing session, each rat was placed in the same closed section. Time spent in and entries into the open sections and transitions of open/closed arm were taken as primary parameters.

### 2.6. Western Blotting

Animals were rapidly sacrificed after anesthesia with chloral hydrate, and the spinal dorsal horn and brains were rapidly removed and frozen on ice. The ACC was identified according to the atlas of Paxinos and Watson [29] and dissected out and quickly frozen in liquid nitrogen. Frozen samples were homogenized with lysis buffer containing a cocktail of phosphatase and proteinase inhibitors and PMSF (Beyotime, Shanghai, China). After denaturation, the lysates were separated on 10% SDS-PAGE gel and transferred to polyvinylidene difluoride PVDF membranes (Bio-Rad, Hercules, CA, USA). The membranes were blocked with 5% nonfat powdered milk in TBST (Tris-buffered saline containing 0.1% Tween 20) for 1 h at room temperature (RT) and then incubated overnight at 4°C with monoclonal rabbit anti-phospho-ERK primary antibody (p-ERK1/2, anti-rabbit, 1 : 2000, in 5% w/v BSA, Cell Signaling, Beverly, MA, USA). After washing in TBST, the membrane was incubated for 1 h at RT with HRP-conjugated goat anti-rabbit antibody (1 : 7500; Bio-Rad), and protein bands were visualized using the Immun-Star HRP Chemiluminescence Kit (Bio-Rad). Images of bands were recorded by the ImageQuant LAS 4000 system (GE Healthcare, Hino, Japan) and the band intensities were quantified using ImageQuant TL software (version 7.0, GE Healthcare). The membranes were then incubated in stripping buffer (0.5 M Tris-HCl [pH 6.8], 10%SDS, and 14.4 mol/L *β*-mercaptoethanol) for 30 min at 50°C and reprobed with monoclonal anti-ERK antibody (total ERK1/2, 1 : 1000; Cell Signaling) as loading controls.

### 2.7. Immunofluorescence

Animals were terminally anesthetized with chloral hydrate and perfused through the ascending aorta with saline followed by 4% paraformaldehyde with 0.01 M PBS (pH 7.2–7.4, 4°C). After perfusion, brains were removed and postfixed in the same fixative for 4–6 h, which was then replaced with 15% and 30% sucrose successively overnight. Brain sections (30 *μ*m) were cut in a cryostat and processed for immunofluorescence. All sections were blocked with 5% goat serum in TBST for 1 h at 37°C and incubated overnight at 4°C with anti-phospho-ERK antibody (p-ERK1/2, rabbit anti-rat, 1 : 400; Cell Signaling). The sections were then incubated for 1 h at 37°C with Cy3-conjugated secondary antibody (1 : 1000; Jackson Immunolabs, West Grove, PA, USA). For p-ERK/NeuN/GFAP/OX-42 double immunofluorescence, sections were incubated with mixture of rabbit anti-p-ERK and mouse anti-NeuN (neuronal marker, 1 : 1000; Abcam, USA), GFAP (astrocytic marker, 1 : 100, Abcam, USA), or OX-42 (microglial marker, 1 : 100, Serotec, Oxford, UK) separately for 16 h at 4°C, followed by a mixture of CY3- and FITC-conjugated (1 : 100, Jackson Immunolabs) secondary antibodies for 1 h at 4°C.

The stained sections were visualized with a Nikon Eclipse Ti confocal microscope (Nikon, Japan) and images were captured with NIS Elements D3.22 software (Nikon).

### 2.8. Statistical Analysis

All data are expressed as mean ± SEM, and statistical analyses were performed using analysis of variance (ANOVA) followed by the least significant difference (LSD) post hoc test with *P* < 0.05 considered significant.

## 3. Results

### 3.1. Pain Hypersensitivity

**Figure 1:**
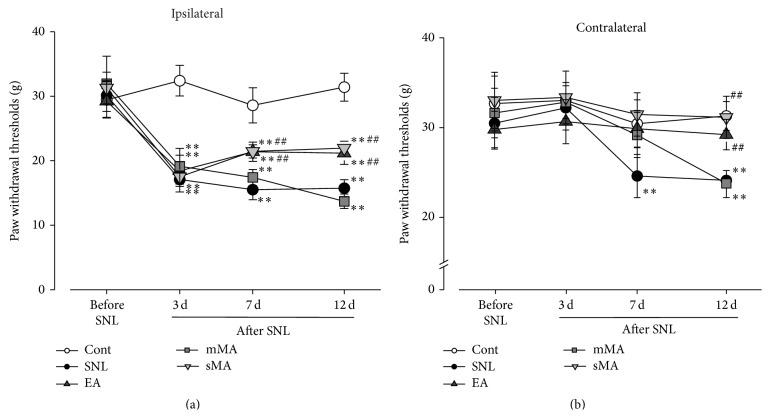
Bilateral changes in nociceptive behaviors after L5 spinal nerve ligation (SNL) in control (non-SNL control, Cont), SNL alone (SNL control, SNL), SNL+ EA stimulation (EA), SNL+ mMA stimulation (mMA), and SNL+ sMA stimulation (sMA) groups. Pain hypersensitivity is measured by ipsilateral and contralateral hind paw withdrawal thresholds (PWTs, g) in response to mechanical stimulation. ^*∗∗*^ *P* < 0.01 versus control group at each time-point; ^##^ *P* < 0.01 versus SNL group at each time-point.

Five groups of rats (non-SNL control (control) and four SNL groups: SNL control (SNL), sMA, mMA, and EA) were tested before surgery and 3, 7, and 12 days afterwards. At 3 d after surgery, the withdrawal threshold of the ipsilateral hind paw showed a profound decrease with control group (Figure 1(a), *P* < 0.01); this had not recovered until 12 d, while sMA and EA intervention, administrated at 3 d after surgery and for continuous 4 treatments with 2 d interval, increased the threshold at days 7 and 12 compared with the SNL rats (Figure 1(a), *P* < 0.01). On the contralateral hind paw, PWTs in the SNL rats decreased at 7 d and 12 d compared with the control group (Figure 1(b), *P* < 0.01). At the same time, PWTs did not decreased in the sMA and EA groups on days 7 and 12 compared with SNL rats (Figure 1(b), *P* < 0.01), with no significant difference between these two groups. PWTs in the mMA group rats decreased at 12 d with control rats (Figure 1(b), *P* < 0.01).

### 3.2. Pain-Induced Anxiety

**Figure 2:**
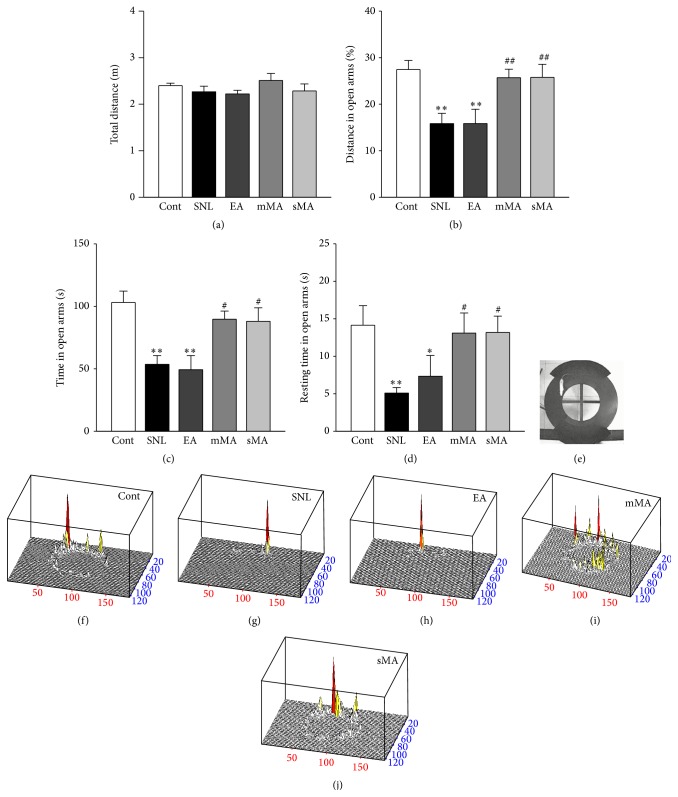
Anxiety-like behaviors in the elevated zero maze during a 5 min test session on day 12 after L5 spinal nerve ligation. (a) Total distance (*F*_(4,28)_ = 1.039, *P* = 0.405); (b) distance in open arms (*F*_(4,28)_ = 5.751, *P* = 0.002); (c) time in open arms (*F*_(4,28)_ = 6.973, *P* = 0.001); (d) resting time in open arms (*F*_(4,28)_ = 3.199, *P* = 0.028); (e) image of detection; ((f)-(g)) analysis graphics of trace. Each column represents the mean ± SEM of six animals per group. ^*∗*^ *P* < 0.05, ^*∗∗*^ *P* < 0.01 versus control group; ^#^ *P* < 0.05, ^##^ *P* < 0.01 versus SNL group.

The elevated zero maze (EZM) is widely used to assess anxiety-like behavior in rodents. It is a modification of the elevated plus maze and has the advantage of lacking the ambiguous central area of the plus maze, resulting in greater sensitivity and reliability. There were no significant difference on total distance in the five groups (Figure 2(a), *F*_(4,28)_ = 1.039, *P* = 0.405). Distance in open arm in SNL rats decreased compared with control rats, and the same happened with EA group (Figure 2(b), *P* < 0.01), while mMA and sMA intervention increased the distance in open arms compared with SNL rats (Figure 2(b), *P* < 0.01). SNL rats spent less total time and resting time in the open sections compared with control rats and so did EA rats (Figures 2(c) and 2(d), *P* < 0.05). Compared with the SNL group, rats in the mMA and sMA groups spent notably more exploratory and resting time in the open sections (Figures 2(c) and 2(d), *P* < 0.05).

### 3.3. Expression of p-ERK in the ACC

**Figure 3:**
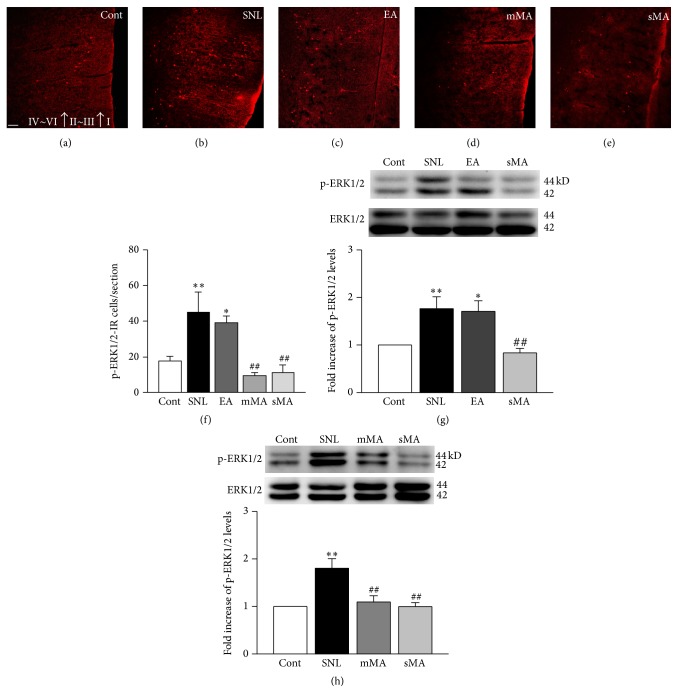
Immunohistochemical ((a)–(f)) and immunoblot ((g)-(h)) analysis of the effect of EA, mMA, and sMA stimulation on p-ERK1/2 protein expression in the anterior cingulate cortex (ACC) after L5 spinal nerve ligation. ((a)–(e)) Expression of p-ERK in the ACC (scale bar = 50 *μ*m; cortical laminae represented by roman numerals); (f) quantification of p-ERK-immunoreactive (IR) cells in the ACC (*F*_(4,10)_ = 8.325, *P* = 0.003); ((g)-(h)) representative blots from ACC homogenates and p-ERK quantification (equal loading was verified by assaying ERK1/2) (*F*_(3,12)_ = 7.418, *P* = 0.005, *F*_(3,12)_ = 9.372, and *P* = 0.002, resp.). Histogram bars represent the mean ± SEM of 5 animals per group. ^*∗*^ *P* < 0.05, ^*∗∗*^ *P* < 0.01 versus control group; ^##^ *P* < 0.01 versus SNL group.

Our immunofluorescence assay revealed an overexpression of p-ERK1/2-positive cells in the ACC of rats on day 12 after surgery, and western blots showed a higher level of p-ERK1/2 protein expression in the SNL group than in non-SNL controls (Figures 3(a), 3(b), and 3(f)–3(h), *P* < 0.01). Interestingly, p-ERK1/2-immunoreactive cell number and protein expression were significantly lower in rats that underwent mMA and sMA stimulation than in SNL rats (Figures 3(b) and 3(d)–3(h), *P* < 0.01). There was no significant difference in p-ERK1/2-immunoreactive cells or protein expression between the EA and SNL groups (Figures 3(b), 3(c), 3(f), and 3(g), *P* > 0.05).

### 3.4. Distribution and Location of p-ERK in ACC Neurons, Macrophages, and Astrocytes

In the control group, p-ERK was expressed largely in laminae II-III (Figure 3(a)), whereas SNL resulted in a wide distribution of p-ERK-immunoreactive cells throughout laminae II–VI (Figure 3(b)). The number of p-ERK1/2-positive cells in the ACC was substantially decreased by mMA and sMA intervention, but their distribution in laminae II–VI was not changed (Figures 3(d) and 3(e)). In comparison with SNL rats, animals that received EA stimulation showed no difference in p-ERK-immunoreactive cell numbers (Figure 3(c)).

**Figure 4:**
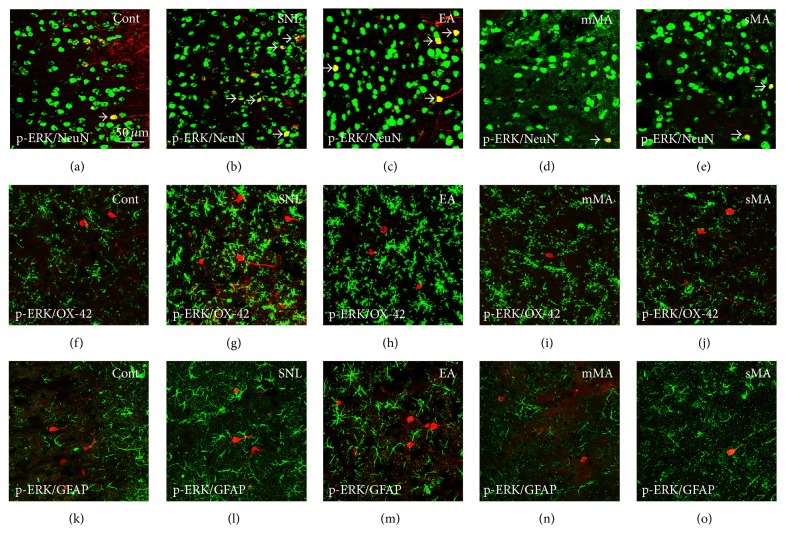
Double immunofluorescence localizing p-ERK in ACC neurons, macrophages, and astrocytes in the ACC after L5 spinal nerve ligation. ((a)–(e)) p-ERK (red) colocalizes with NeuN (green); ((f)–(j)) p-ERK (red) does not colocalize with OX-42 (green); ((k)–(o)) p-ERK (red) does not colocalize with GFAP (green).

**Figure 5:**
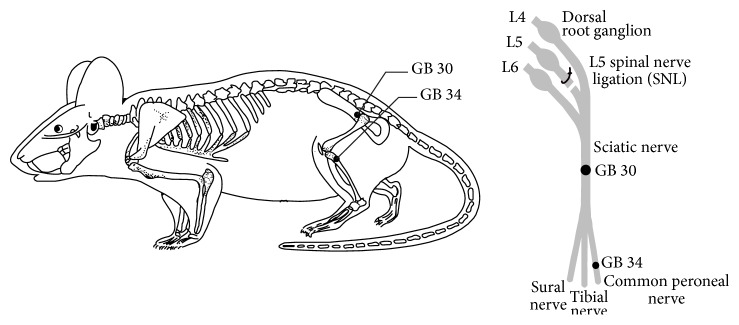
Schematic representation of the GB 30 and GB 34 in the rat.

Very few p-ERK-immunoreactive neurons were found in the ACC in non-SNL control rats (Figure 4(a)). SNL induced an immediate activation of ERK in ACC neurons (Figure 4(b)). No visual difference in p-ERK/NeuN double-labeled neurons was observed in the ACC of rats that received EA compared with SNL (Figures 4(b) and 4(c)). Compared with SNL, mMA, and sMA stimulation decreased p-ERK/NeuN double-labeled neurons (Figures 4(b), 4(d), and 4(e)). We found no p-ERK/OX-42 or p-ERK/GFAP double-labeled macrophages or astrocytes (Figures 4(f)–4(o)).

## 4. Discussion

The present study is the first to show that both sMA and EA stimulation significantly relieve mechanical hypersensitivity in a rat model of neuropathic pain, and both sMA and mMA stimulation significantly reduce anxiety-like behavior. The changes in anxiety-like behavior correlate with ERK activation in ACC neurons. These results extend our previous data showing that p-ERK in the bilateral spinal horn is involved in sMA inhibition of SNL-induced bilateral mechanical hyperalgesia [30]. Hence, sMA might be the treatment of choice in cases of neuropathic pain accompanied by anxiety.

The International Association for the Study of Pain (IASP) defines pain as “an unpleasant sensory and emotional experience associated with actual or potential tissue damage or described in terms of such damage” [31]. It denotes that the experience of pain comprises a sensory dimension and an affective dimension. Pain-induced negative sequelae can be more disabling than the pain itself, severely affecting the daily activities of patients with persistent pain [5]. Many studies on persistent pain have paid considerable attention to its effects on anxiety-like behavior [32, 33]. It is necessary to distinguish the sensory and affective changes in pain treatment. SNL is a common model for mechanical hyperalgesia and pain-induced anxiety [34, 35]. Animals with SNL have enhanced escape/avoidance behavior 3 days following the lesion [36]. Anxiogenic-like behaviors have also been observed in the light/dark test up to 4 weeks after SNL [37, 38].

Acupuncture is an important component of traditional Chinese medicine (TCM) and a practical science of preventing and treating diseases. Needling technique means the methods through stimulating certain parts of human body (acupoints) by means of different kinds of needles or nonneedle methods with certain manipulation techniques [39]. Manual acupuncture (MA) is the insertion of an acupuncture needle into acupoints followed by the twisting of the needle with different manual strength, and electroacupuncture (EA), a stimulating current via the inserted needle, is delivered to acupoints [40]. As a clinical acupuncturist, it is a key factor to select optimal needling techniques based on the disease condition of patient.

In clinical application of acupuncture, GB 30 is always the main point or the basic point in the low back pain and GB 34 is used as an additional point when low back pain is associated with the numbness and pain of lower extremities [39, 41]. The L5 spinal nerve ligation-induced neuropathic pain is similar to the low back pain in clinical. Moreover, in anatomy, L4, L5, and L6 spinal nerve were combined into the sciatic nerve, which is divided into common peroneal nerve, tibial nerve, and sural nerve. GB 30 locates on the sciatic nerve path and GB 34 situates on the common peroneal nerve. Therefore, in this study we select GB 30 (main point) and GB 34 (additional point) to alleviate SNL-induced neuropathic pain.

Analgesia with manual acupuncture or EA is essentially a manifestation of integrative processes at the dorsal root ganglion and central nervous systems between the afferent impulses from the pain regions and acupoints [42–45]. Researchers and clinicians have increasingly focused on EA analgesia [46, 47], for its feasibility in developing a standardized treatment protocol for analgesia; this approach is supported by our results, in which sMA and EA treatments had equivalent effects on pain. However, clinical evidence has not been conclusive on the effects of manual acupuncture or EA on anxiety [18], despite the fact that both are increasingly used for the treatment of anxiety. In basic research, a few studies examining the effects of manual acupuncture and EA on anxiety have indicated that the technique might reduce anxiety-like behavior in adult rats [48–50]. Our early results indicated that sMA, but not mMA, could alleviate SNL-induced bilateral pain which is closely related to its effect in downregulating the expression of p-ERK in the bilateral spinal dorsal horn regions [30]. In the present study, we observed that the sensory dimension of neuropathic pain, measured by PWTs, decreased consistently from day 3 after surgery. The rat's bilateral mechanical hypersensitivity was significantly inhibited by either sMA or EA stimulation on 12 days after SNL, but not mMA. We also explored the affective dimension with EZM tests. Twelve days after SNL, rats displayed increased anxiety behavior. Interestingly, sMA and mMA stimulation alleviated pain-induced anxiety in the EZM test, whereas EA stimulation was without effect. These results indicate that sMA is considerably more effective than mMA and EA in relieving both mechanical hypersensitivity and concomitant anxiety. The differential effects may reflect a difference in afferent activation in the supraspinal pathway, especially in cerebral nuclei.

To examine this possible mechanism underlying the effects of sMA stimulation on pain-induced anxiety, we explored p-ERK expression in the ACC. Peripheral nerve injury has been reported to induce neuroplastic changes in different regions including the insular cortex, amygdala, and ACC, which have been associated with pain-like aversive behaviors and depressive-like symptoms [34, 51, 52]. Results from numerous human and animal studies indicated that the ACC, which forms one of the largest parts of the limbic system, plays an important role in the affective component of pain [20, 43, 53]. Extensive studies supported the notion that the ACC is a pivotal region for emotion [22]. Another study reported that electrical stimulation of the ACC attenuates the aversive quality of noxious cutaneous hind paw stimulation without producing an antiallodynia effect in rats with SNL [54]. These suggested that the ACC regulates affective pain processing.

ERK activation in the ACC was required for the expression of pain-induced anxiety [27]. Another study hypothesized that attenuation of p-ERK1/2 overactivity in the ACC represented a potentially valuable therapeutic strategy for the relief of pain-induced anxiety [28]. A previous report suggested that ERK cascades in the ACC are involved in pain-induced negative emotion in the rat [55]. ERK also played a crucial role in persistent pain-enhanced temporal synaptic plasticity [56]. In our study, ERK1/2 activation in the ACC of rats after SNL was significantly stronger than in non-SNL control rats, and this coincided with changes in anxiety-like behavior. Accordingly, sMA and mMA stimulation downregulated ERK1/2 activation in ACC neurons and also improved anxiety-like behavior, suggesting a mechanism behind the effect of sMA and mMA on pain-induced anxiety.

Differences in stimulation strength and method may lead to differences in treatment results; the parameters used in manual acupuncture and EA when treating anxiety are not yet clear and are worthy of further study. It is also important to evaluate the present findings in a clinical setting.

In summary, sMA stimulation may relieve both mechanical hyperalgesia and pain-induced anxiety in a rat model of neuropathic pain, while mMA only reduces anxiety and EA only alleviates mechanical hypersensitivity. We propose that sMA could be a two-dimensional Chinese medicine therapy on the sensory and affective dimension of pain. The effect of different acupuncture stimulation on anxiety-like behavior may arise from the regulation of p-ERK in ACC neurons.

## Figures and Tables

**Table 1:** The comparison of three different acupuncture stimulation groups.

Stimulation patterns	Stimulation parameters	Acupoints	Time of treatment
EA	Constant square wave current output (pulse width: 0.6 ms at 2 Hz, 0.2 ms at 100 Hz); remaining intensities at 1 ± 0.5 mA (causing the slight vibration of muscles around acupoints); alternating frequencies of 2 Hz and 100 Hz (automatically shifting between 2 Hz and 100 Hz stimulation for three seconds each).		
sMA	Sterilized disposable stainless steel needles (0.3 mm in diameter) on bilateral GB 30 were inserted to a depth of 10 mm and then twisted manually clockwise and counterclockwise (360°) for 2 min at a rate of 180 times per min, followed by an interval of 13 min with needles retained and then another 2 min twisting stimulation. Finally the needles were retained in place for 13 min before removal. The bilateral GB 34 simply retained the needles for 30 min without any twirling and rotating manipulation.	The main point is GB 30 and the additional point is GB 34.	30 min
mMA	Sterilized disposable stainless steel needles (0.22 mm in diameter) on bilateral GB 30 were inserted to a depth of 10 mm and then twisted manually clockwise and counterclockwise (180°) for 2 min at a rate of 60 times per min, followed by an interval of 13 min with needles retained and then another 2 min twisting stimulation. Finally the needles were retained in place for 13 min before removal. The bilateral GB 34 simply retained the needles for 30 min without any twirling and rotating manipulation.		
